# On the pitfalls of conceptualizing excessive physical exercise as an addictive disorder: Commentary on Dinardi et al. (2021)

**DOI:** 10.1556/2006.2022.00001

**Published:** 2022-03-01

**Authors:** Damien Brevers, Pierre Maurage, Taylor Kohut, José C. Perales, Joël Billieux

**Affiliations:** 1 Psychological Sciences Research Institute, UCLouvain, Louvain-la-Neuve, Belgium; 2 Institute for Health and Behaviour, Department of Behavioural and Cognitive Sciences, University of Luxembourg, Esch-sur-Alzette, Luxembourg; 3 Mind, Brain, and Behavior Research Center (CIMCYC), Department of Experimental Psychology, University of Granada, Granada, Spain; 4 Institute of Psychology, University of Lausanne, Lausanne, Switzerland; 5 Centre for Excessive Gambling, Addiction Medicine, Lausanne University Hospitals (CHUV), Lausanne, Switzerland

**Keywords:** physical exercise, self-determination theory, addiction-based approach, process-based approach, debate

## Abstract

This commentary challenges some of the proposals made in the opinion paper entitled “The expanded interactional model of exercise addiction” by Dinardi, Egorov, and Szabo (2021). We first question the usefulness of the (expanded) interactional model of exercise addiction to determine the psychological processes underlying distress and functional impairment in excessive physical exercise. We then consider the authors’ use of the Self-Determination Theory to model exercise addiction, which risks the misclassification of strenuous, but adaptive, patterns of physical exercise as exercise addiction. We finally address broader concerns regarding the idea that maladaptive exercising could be conceptualized as an addictive disorder.

In a recent opinion paper, Dinardi, Egorov, and Szabo (2021) present a theoretical model aimed at “*conceptualizing exercise addiction as a disorder with unique antecedents, contributing factors, and consequences that set it apart from other dysfunctions*” in order to “*generate more precise ideas about what exercise addiction is and how to assess it*” (Dinardi et al., 2021, p. 627). This paper updates and expands their interactional model of exercise addiction (Egorov & Szabo, 2013), wherein “exercise addiction” constitutes a condition in which intensive involvement in exercise behavior negatively interferes with various life areas (e.g., health, social and affective relationships, work/school performance). While we acknowledge that intensive exercise can be severely problematic for some vulnerable people in specific circumstances, we are not convinced that this expanded interactional model of exercise addiction can usefully inform our understanding of this phenomenon. We elaborate here on four reasons to question Dinardi et al.’s (2021) proposal that maladaptive exercise is best conceptualized as an addictive disorder, or even as a diagnosable mental disorder.

## Inaccurate use of the Self-Determination Theory risks pathologizing intensive physical exercise

The update proposed by Dinardi and colleagues (2021) focused on the “*factors that lead an individual to be interested in exercise as an outlet for physical activity, their specific motivations, and orientations that describe their approaches in using exercise and sports to experience mastery, enhance specific elements of their life, and as means of stress-coping.*” (Dinardi et al., 2021, p. 627). In order to reach this goal, the authors mainly capitalized on the influential Self-Determination Theory (SDT; Deci & Ryan, 1985, 2000; Ryan & Deci, 2017). Existing evidence suggests that, despite its positive links with introjected (guilt-based) regulation, addiction to physical exercise is positively associated with self-determined motivations toward physical exercise (identified, integrated, and intrinsic regulation), as well as with the fulfilment of a basic psychological need for competence. This pattern is robust across samples recruited in fitness centers (i.e., “regular exercisers”) and in high-performance training centers (i.e., “athletes”; Edmunds, Ntoumanis, & Duda, 2006b; González-Cutre and Sicilia, 2012; Hamer, Karageorghis, & Vlachopoulos, 2002; Kovácsik, Tóth-Király, Egorov, & Szabo, 2021; Sicilia, Alcaraz-Ibáñez, Lirola, Burgueño, & Maher, 2018; Symons Downs, Savage, & DiNallo, 2013; but see Costa, Hausenblas, Oliva, Cuzzocrea, & Larcan, 2016; Tornero-Quiñones, Sáez-Padilla, Castillo Viera, García Ferrete, & Sierra Robles, 2019). In other words, the higher the level of exercise addiction, the higher the level of self-determination toward physical exercise (see also Szabo, 2018). These findings contrast with studies showing robust associations between overtraining or sports burnout and reduced self-determination (i.e., lower levels of intrinsic regulation, integrated regulation, and identified regulation and higher levels of amotivation, external regulation, and introjected regulation), as well as lower satisfaction of basic psychological needs (for a narrative review, see Groenewal, Putrino, & Norman, 2021; for a systematic review and meta-analyses, see Li, Wang, Pyun, & Kee, 2013; for recent studies, see De Francisco, Sánchez-Romero, Vílchez Conesa, & Arce, 2020; Fagundes, Noce, Albuquerque, de Andrade, & Teoldo da Costa, 2021).

In our opinion, the existing evidence challenges the proposal made by Dinardi et al. (2021), namely conceptualizing key SDT dimensions as promoting physical exercise addiction. More specifically, the way that Dinardi et al. (2021) used SDT to model exercise addiction suffers from a high risk of misclassifying strenuous, but adaptive, patterns of physical exercise as addictive. This risk is exemplified by two studies carried out by Edmunds, Ntoumanis, and Duda (2006a, 2006b) among comparable samples of regular exercisers. In the first study, these authors examined self-determination levels in relation to addictive exercise involvement and found a more pronounced psychological need for exercise-related competence and higher degrees of external, introjected, identified, integrated, and intrinsic regulations in individuals assessed as being prone to exercise addiction (Edmunds et al., 2006a). In the second study, they framed strenuous physical activity as health promoting and unveiled similar patterns of positive associations between introjected, identified, and intrinsic degrees of self-determination, on the one hand, and indexes of strenuous physical exercise on the other (Edmunds et al., 2006b). Strikingly, the two sets of results are interpreted in opposite ways: When framing strenuous physical exercise as indexing addictive disorder (Edmunds et al., 2006a), the authors interpreted the results as evidence that *“SDT could be considered in the development of inventories to assist the successful diagnosis of problematic exercise engagement. Interventions designed to* *support* *individuals displaying exercise dependence symptomatology may also benefit from being grounded in SDT […] health and exercise professionals who focus upon the promotion of psychological need satisfaction and self-determined forms of motivation”* (Edmunds et al., 2006a, p. 900). In contrast, when strenuous physical exercise was framed as a health-promoting behavior (Edmunds et al., 2006b), the results were interpreted as supporting the usefulness of SDT in explaining healthy and harmonious involvement in physical exercise.

What these studies actually demonstrate is that SDT dimensions are predictive of commitment to exercise, which is in turn linked to more intensive and strenuous physical activity in regular exercisers and athletes, as well as to higher exercise addiction symptoms as measured by available scales. However, the fact that those links do not vary according to the adaptive or maladaptive nature of strenuous exercise also suggests that SDT dimensions are not specifically predictive of disordered aspects of exercise involvement, conceptualized as an addictive disorder. Consequently, incidence and prevalence rates of exercise addiction are likely inflated by misclassifying committed sportspeople who use exercise to attain their personal needs as “addicted.” Such conceptual problems are also related to measurement issues, discussed in the following section.

## Validity problems in exercise addiction measurement

Although Dinardi et al.’s did not discuss the assessment of exercise addiction, their model is derived from evidences obtained through questionable but widely used exercise addiction scales. Most scales that assess physical exercise addiction have capitalized on a potentially flawed confirmatory approach, recycling and adapting substance use disorder criteria (for a critical account of the confirmatory approach in behavioral addiction research, see Billieux et al., 2015; Kardefelt-Winther et al., 2017). To the extent that such scales are prone to false positives (i.e., pathologizing intensive but healthy patterns of physical exercise), scores obtained on these scales will spuriously correlate with adaptive traits (as described in the previous section), wrongly suggesting that such adaptive traits contribute to addiction. We believe that this may be one of the reasons that SDT fails to discriminate between adaptive and maladaptive exercise behaviors.

For example, one of the most popular assessment instruments in this field is the six-item Exercise Addiction Inventory (EAI; Terry, Szabo, & Griffiths, 2004), in which tolerance (as an addiction feature) is measured through the following single item: “Over time I have increased the amount of exercise I do in a way.” While logical for substance-related addiction, such an item is irrelevant in sport, as it most likely measures nothing more than healthy progression or the mere training effect (e.g., someone becoming able to run longer as they progressively improve their physical condition; see also Szabo, Griffiths, deLa Vega Marcos, Mervó, & Demetrovics, 2015). As noted earlier, as long as exercise addiction scales contain items that do not discriminate problematic from non-problematic commitment, elevated scores on such scales will be severely inflated and contaminated by invalid items that are not able to capture significant levels of psychological distress.

Specifically, in relation to criterion and convergent validity, the cutoffs proposed for the three most frequently used exercise addiction scales (the Exercise Dependence Scale [EDS], Hausenblas & Downs, 2002; the EAI, Terry et al., 2004; and the Compulsive Exercise Test [CET], Tanaris, Touyz, & Meyer, 2011) were established by recruiting individuals (mostly college students) who exercise or play sports regularly. In the case of the EDS, no specific information was even provided on the level of physical exercise involvement required to be included. Moreover, the studies on the EAI and the CET did not include any measures of psychological distress; in contrast, the study for the EDS included measures of mood states and state-trait anxiety, but no significant association with EDS scores was observed. However, these validation studies did report positive correlations between physical exercise addiction symptoms and eating disorder symptoms (for the EDS and CET; the EAI did not include such measures). It is thus likely that even when what is measured by such scales is genuinely problematic, it is impossible to ascertain that what is measured is not just a potentially maladaptive strategy displayed to cope with symptoms of an eating disorder (Bamber, Cockerill, Rodgers, & Carroll, 2000, 2003; Coniglio, Cooper, & Selby, 2021). Such an account is in line with data showing that the association between physical exercise addiction and reduced quality of life does not hold after statistically controlling for the effects of eating disorder psychopathology (Mond, Hay, Rodgers, & Owen, 2006).

## The primary versus secondary disorder fallacy

While we commend Dinardi and colleagues (2021) for their focus on the psychological processes underlying maladaptive involvement in physical exercise (as behavioral addiction research too often favors symptom-based over process-based approaches; see Billieux et al., 2015; Perales et al., 2020), we believe that the conceptual framework proposed ensnares its understanding by applying self-limiting conceptualizations and non-falsifiable arguments. To be more precise, when detailing the distinction between primary and secondary exercise addiction, Dinardi et al. (2021) interpret anxiety and stress coping as markers of both primary (“*using exercise and sports to experience mastery, enhance specific elements of their life, and as means of stress-coping*,” p. 627) and secondary (*“exercise behavior that is foremost motivated by the desire to relieve anxiety people experienced specifically as a result of not exercising*,*”* p. 627) disorders. The authors also provide what we consider to be confusing arguments when elaborating how the expanded interactional model may account for the fact that the way “*people see and think about themselves could be a mediator of maladaptive exercise*” (p. 628). Indeed, Dinardi et al. (2021) referred to a study that examined body dysmorphic disorder and other image-related psychopathological correlates in fitness (Corazza et al., 2019). This pattern characterizes a situation in which addictive involvement likely results from maladaptive coping that is displayed in order for the individual to face primary psychological problems and thus corresponds to the psychiatric conceptualization that the authors want to depart from.

In more general terms, we believe the primary/secondary exercise addiction distinction is arbitrary, as all behaviors maintained by negative reinforcement are “secondary” to the “primary” process by which the consequences to be avoided are experienced as aversive. Accordingly, exercise involvement can still be induced by body weight-related concerns (e.g., to run more often and more intensely when somebody feels unhappy by their gained weight) despite the absence of clinically relevant symptoms of eating disorder. Indeed, scores of eating or body-related concerns remain high in samples who are supposed to have a “primary addiction” to physical exercise (e.g., Grandi, Clementi, Guidi, Benassi, & Tossani, 2011). Nevertheless, numerous studies that focus on primary exercise addiction used cutoff scores for excluding individuals with eating disorders and body-related concerns (e.g., Costa et al., 2016), rather than including these variables as covariates of interest, thus potentially including individuals with subclinical levels of eating disorders or body-related concerns.

## Links between specific motives and distinct forms of physical activity

Although it is indeed important to consider the various motives underlying intense (either healthy or problematic) physical exercise, we believe it is not tenable to associate specific motives with distinct activities, as done by Dinardi et al. (2021) when they claim that fitness is driven by a desire to enhance one’s physical appearance or health, whereas sport participation is driven by performance. In fact, the pluridimensional aspect of physical exercise-related motives was demonstrated by Szabo (2018) in a case study of an adult involved in bodybuilding: Simply categorizing individuals as fitness or sport exercisers is often not even feasible (Deelen, Ettema, & Kamphuis, 2018; Szabo, 2018). Perhaps even more problematic is the fact that the proposed model itself does not inform on how distinct motivational patterns differentially lead to maladaptive physical exercise. This issue becomes evident in the last three subsections of Dinardi et al.’s (2021) paper. The sections “Personal and Situational Factors” and “Incentives for Exercise” mention relevant constructs (e.g., self-concept), but we believe they constitute core processes underpinning sport involvement rather than potential markers of an addictive disorder. In particular, the section devoted to “exercise-related stressors” does not specify how the addiction framework is relevant in accounting for the association between stress, anxiety, and maladaptive involvement in physical exercise.

The conceptual framework provided would thus predict generic and unspecific pathways to excessive physical exercise. For instance, fear of failure may lead to overinvolvement in physical exercise (Taylor, Eklund, & Arthur, 2021), but it may also similarly and unspecifically relate to drug use (e.g., Blank, Schobersberger, Leichtfried, & Duschek, 2016), inadequate training habits (e.g., trying to compensate for anxiety-related underperformance via overtraining), staleness, burnout, or injury; it can also fuel anxiety itself (for a scoping review, see Taylor et al., 2021). It is thus unclear to us how the (expanded) interactional model of exercise addiction could offer an innovative and fine-grained understanding of the vicious cycle related to, for example, performance anxiety. Ultimately, these issues signal a major limitation in adapting the addiction framework to excessive physical exercise in order to develop tailored and individualized prevention and treatment strategies.

## Concluding thoughts

We agree with Dinardi and colleagues (2021) that maladaptive involvement in physical exercise should be examined by using an interactional dynamic approach, but probably not within the proposed framework anchored in the addiction model. The large influence of this model on current research (illustrated by the number of citations related to the original model, as repeatedly mentioned by the authors) could constitute an opportunity to renew this theoretical approach. This could notably be done by revisiting this model through process-based (e.g., Kinderman & Tai, 2007; Philippot, Bouvard, Baeyens, & Dethier, 2019) or network-based approaches (e.g., Robinaugh, Hoekstra, Toner, & Borsboom, 2020) in order to consider the holistic and dynamic links between psychological distress and overinvolvement in physical activity, that is, not as a “primary” or “secondary” disorder (e.g., Billieux et al., 2015).

More specifically, one could identify the psychological processes (e.g., cognitive distortions resulting from maladaptive perfectionism; negative body image linked to low self-esteem) through which physical activity and other behaviors (e.g., eating habits) trigger short-term reinforcing effects (e.g., sense of control over the body) while maintaining the long-term negative consequences of a psychological problem. This approach should also be fostered by the use of process-oriented measures for indexing physical activity habits. For instance, Grove, Zillich, and Medic (2014) developed a self-report measure that taps into patterned action (e.g., “I exercise at the same location each week”), stimulus-response bonding (e.g., “Certain surroundings just make me want to exercise”), automaticity (e.g., “I exercise without conscious reminders to do so”), and negative consequences if not done (e.g., “If I don’t exercise, I feel irritable”) as indicators of the strength of physical activity habits, rather than a marker of the severity of an addictive disorder. Such a processual approach appears to be more adapted than the addiction-focused approach to developing theoretical knowledge on maladaptive exercise, and hence developing adapted therapeutic proposals.

## Funding sources

The work of D.B. is funded by the Luxembourg National Research Fund (FNR); CORE – Junior Track [BETHAB; C18/BM/12552025]. J.C.P. is supported by a grant by the Spanish Research Agency (Agencia Española de Investigación), Spanish Ministry of Science and Innovation (Ministerio de Ciencia e Innovación) (MCIN/AEI/10.13039/501100011033/), with reference PID2020-116535GB-I00. P.M. (Senior Research Associate) is funded by the Belgian Fund for Scientific Research (F.R.S.-FNRS, Belgium). These funds did not exert any editorial direction or censorship on any part of this article.

## Author’s contribution

D.B., J.C.P., and J.B. wrote the original draft of the manuscript. All authors significantly contributed to and have approved the final manuscript.

## Conflict of interest

All authors declare no conflicts of interest relevant to this manuscript.
